# Size at birth predicts later brain volumes

**DOI:** 10.1038/s41598-023-39663-9

**Published:** 2023-08-01

**Authors:** Samson Nivins, Eleanor Kennedy, Christopher McKinlay, Benjamin Thompson, Jane E. Harding, Children with HYpoglycemia and Their Later Development (CHYLD) Study Team, Jane Alsweiler, Gavin Brown, Gregory Gamble, Trecia Wouldes, Peter Keegan, Deborah Harris, Geoffrey Chase, Jason Turuwhenua, Jenny Rogers, Rajesh Shah, Darren Dai, Jocelyn Ledger, Stephanie Macdonald, Alecia McNeill, Coila Bevan, Nataliia Burakevych, Robin May, Safayet Hossin, Grace McKnight, Rashedul Hasan, Jessica Wilson, Jennifer Knopp, Arijit Chakraborty, Tony Zhou, Steven Miller

**Affiliations:** 1 Liggins Institute, University of Auckland, Building 503, Level 2, 85 Park Road, Auckland, New Zealand; 2 Kidz First Neonatal Care, Counties Manukau Health, Auckland, New Zealand; 3 School of Optometry and Vision Science, University of Waterloo, Waterloo, ON Canada; 4 Centre for Eye and Vision Research, The Hong Kong Polytechnic University, 17W Science Park, Shatin, Hong Kong; 5 University of Auckland, Auckland, New Zealand; 6 Victoria University of Wellington, Wellington, New Zealand; 7 University of Canterbury, Christchurch, New Zealand; 8 University of British Columbia, Vancouver, Canada

**Keywords:** Health care, Medical research, Neurology

## Abstract

We aimed to investigate whether gestation at birth, birth weight, and head circumference at birth are still associated with brain volume and white matter microstructure at 9–10 years in children born late-preterm and at term. One hundred and eleven children born at ≥ 36 weeks gestation from the CHYLD Study cohort underwent brain magnetic resonance imaging at 9 to 10 years. Images were analysed using FreeSurfer for volumetric data and tract-based spatial statistics for diffusion data. Of the cohort, 101 children were included for volumetric analysis [boys, 49(49%); median age, 9.5 (range: 8.9–12.4) years]. Shorter gestation at birth, lower birthweight, and smaller birth head circumference were associated with smaller brain volumes at 9 to 10 years, both globally and regionally. Amongst the perinatal factors studied, head circumference at birth was the strongest predictor of later brain volumes. Gestation at birth and absolute birthweight were not associated with diffusion metrics of white matter skeleton. However, lower birthweight z-score was associated with higher fractional anisotropy and lower radial diffusivity. Our findings suggest that even in children born late preterm and at term, growth before birth and timing of birth are still associated with brain development in mid-childhood.

## Introduction

Fetal brain development is a complex process that occurs continuously throughout pregnancy. During the third trimester, brain size increases four-fold, accompanied by the formation of tertiary sulci and gyri^1^. Preterm birth interrupts this pattern of normal brain development in the in-utero environment, and is associated with smaller brain volumes and altered white matter microstructure during early childhood and adolescence^2,3^. For example, children born late preterm (34 to 36 weeks) had smaller volumes of the total tissue (i.e., cerebral cortex and cerebral white matter) and thalamus compared with their term-born peers at 6 to 13 years^2^. Size at birth is also related to brain volumes throughout childhood and adolescence in children born preterm^4^. However, to our knowledge, few studies have explored these relationships in those born at or near term^5,6^. Furthermore, it is unclear whether slower growth before birth, leading to lower birth weight, the timing of birth, or both, is important for later brain growth, and little is known about the relationships between gestation or size at birth and white matter microstructure during childhood.

Head circumference is a commonly used indicator of brain size during infancy and early childhood, and there is a weak association between head circumference during childhood and concurrent brain volumes^7^. One study has reported a positive association between head circumference at birth and total brain volume at ten years in children born at term^8^.

Boys are at increased risk of preterm birth and have larger head circumference than girls from the second trimester onwards^9^. The impact of preterm birth on later brain growth is also greater in boys than girls; but it is not known whether there are any sex differences in the relationship between size at birth and later brain development.

In this study, we aimed to investigate the relationships between gestation at birth, birthweight, and head circumference at birth, and brain volume and white matter microstructure in school-aged children born late-preterm and at term using magnetic resonance imaging (MRI). We hypothesised that children born at shorter gestation, at lower birth weight, and with a smaller head circumference at birth would have smaller brain volumes and also would have altered white matter maturation at 9 to 10 years. Additionally, we investigated whether these relationships differ in boys and girls.

## Methods

The Children with HYpoglycaemia and their Later Development (CHYLD) study is a prospective cohort investigation of moderate to late preterm and term neonates born from December 2006 to November 2010 at Waikato Hospital, Hamilton, New Zealand at ≥ 32 weeks’ gestation and with one or more risk factors for neonatal hypoglycaemia [i.e., maternal diabetes, preterm, low birth weight (< 10th percentile or < 2500 g), or high birth weight (> 90th percentile or > 4500 g)]^10,11^. Infants with serious congenital malformations or terminal conditions were excluded^10,11^.

All surviving children who had not previously withdrawn were invited to undergo a neurodevelopmental assessment at 9 to 10 years’ corrected age between 2018 and 2020. Details can be found elsewhere^12^.

For this MRI study, only children born at ≥ 36 weeks’ gestation were eligible to avoid the confounding effect of prematurity on brain development. The severity of neonatal hypoglycaemia experienced was grouped as none (no evidence of any hypoglycaemia), mild (1 to 2 hypoglycaemic events 2.0 to 2.6 mM), severe (any hypoglycaemia events < 2 mM or ≥ 3 hypoglycaemic events), or clinically undetected hypoglycaemia (interstitial episodes only)^13^. Up to 75 eligible children in each hypoglycaemia group were randomly selected using random number tables by a staff member who was not involved in the MRI study. Parents of these randomly selected children were asked for consent to be approached about the MRI study at the time of seeking consent for the neurodevelopmental assessment. Those who consented were then approached in order of the children’s dates of birth until there were no further families who had agreed to be approached in the group, or until 30 MRI studies had been completed in each group (Supplemental Fig. 1).

The effects of neonatal hypoglycaemia on brain outcomes and white matter microstructure are described elsewhere^13^, and are not explored in this paper. The complete study was approved by the Health and Disability Ethics Committee (16/NTB/208/AM02), the parents or legal guardian gave written informed consent, and the child gave verbal assent. All methods were carried out in accordance with relevant guidelines and regulations.

### Image acquisition and processing

The T1-weighted structural (0.8mm^3^ isotropic voxel resolution, slice thickness = 0.85 mm, repetition time (T_R_) = 2000 ms, echo time (T_E_) = 3.51 ms, field of view (FoV) = 210 mm, 176 slices, and flip angle 9°) and diffusion-weighted images (64 diffusion sensitising gradients, b = 0 and b = 2000 s/mm^2^, slice thickness = 2.3 mm, T_R_ = 8500 ms, T_E_ = 112 ms, FoV = 225 mm, and 58 slices) were acquired between 2018 and 2020 using 3 T scanner (Magnetom Skyra; Siemens, Erlangen, Germany) equipped with a 20-channel head coil.

All structural imaging data were processed using the software tool: FreeSurfer (version 6.0; http://surfer.nmr.mgh.harvard.edu/). The complete cortical and subcortical reconstructions of the T1-weighted images were performed using the ‘recon all’ script, as described elsewhere^14,15^. Briefly, the procedure includes the removal of non-brain tissue using the watershed algorithm, automated Talaraich transformation, segmentation of the subcortical white and deep grey matter-volumetric structures, intensity normalisation, tessellation of the grey/white matter boundary, automated topology correction, and surface deformation. Then, the images were registered to a spherical atlas based on individual cortical folding patterns to match cortical geometry across participants, and the cerebral cortex was parcellated into 34 regions per hemisphere based on the sulci and gyri^16^. For the volumetric measures, global (total brain, total cortical grey matter, total cerebral white matter, and subcortical grey matter); regional (frontal, parietal, temporal, and occipital lobes), cerebellum, and brainstem were considered. For regional and cerebellum measures, both the left and right sides were combined together.

The diffusion-weighted images were quality controlled and processed with the FSL diffusion toolbox (FSL; http://www.fmrib.ox.ac.uk/fsl, Oxford, UK)^17^. Images were corrected for eddy currents and head motion, and non-brain tissue was stripped. Following quality control of the diffusion-weighted images, the diffusion toolbox was used to fit the tensor model at each voxel and compute fractional anisotropy (FA), axial diffusivity (AD), mean diffusivity (MD), and radial diffusivity (RD) maps.

FA maps from every participant were further processed using tract-based spatial statistics (TBSS) in FSL^18^. The FA maps from each participant were registered to each other to identify the most representative target image using linear registration and non-linear wrapping. The target image was selected based on the lowest mean displacement across all the participants. The chosen target image was aligned into Montreal Neurological Institute 152 standard space, and a study-specific mean FA map was generated, then thinned to create a mean FA skeleton, representing the centre of white matter tracts common to the whole cohort. The skeleton was thresholded to include white matter voxels with FA values > 0.2 to avoid inter-subject variability and to exclude voxels that were either grey matter or cerebrospinal fluid^18^. Individual participants’ aligned FA maps were projected onto this skeleton, and the resulting data were analysed using voxel-wise cross-group statistical analysis with a non-parametric approach utilising permutation testing with a standard general linear model approach using ‘randomise’. The locations of significant clusters were determined using the John Hopkins University ICBM-DTI-81 white matter tractography atlas tools integrated into the FSL software package. The other diffusion metrics: MD, AD, and RD, were similarly extracted and projected onto the FA skeleton, and the resultant maps were analysed as above.

### Statistical analysis

Birth weight and absolute head circumference at birth were obtained from clinical records. Birth weight was measured naked using calibrated electronic scales to the nearest 10 g. Birth head circumference was measured at the maximal occipitofrontal circumference with a non-stretchable tape to the nearest 0.1 cm. Z-scores were calculated using New Zealand population reference ranges^19^. The socio-economic decile was derived from the New Zealand deprivation index (NZDPI), where 10 indicates the most and 1 the least deprived^20^. Baseline demographic characteristics and clinical measurements were compared between groups using the Students’ t-test and one-way analysis of variance for continuous data and Pearson’s Chi-square or Fisher’s exact tests for categorical data. Linear regression analyses were used to identify the relationships between gestation at birth, birth weight, head circumference at birth and global and regional brain volumes. As this was an exploratory study, we did not perform any adjustments for multiple comparisons.

Additionally, to investigate the independent effects of perinatal factors on brain volumes, we created two models: Model 1 where both gestation at birth and birthweight z-score were included, and Model 2 where both gestation at birth and head circumference z-score were included.

For the diffusion data, generalised linear models were used to assess the relationships between gestation at birth, birth weight, and diffusion metrics (FA, MD, AD, and RD) in TBSS using FSL’s ‘randomise’ permutation tool. The results were considered significant if *p* ≤ 0.05, corrected for multiple comparisons using family-wise error, and thresholded with threshold-free cluster enhancement option^18^. All statistical analyses were performed using JMP version 14.0 (SAS Institute Inc., North Carolina, USA), except for diffusion data which was analysed using FSL.

## Results

Of the 248 eligible children, the parents of 170 agreed to be approached about MRI, and 111 children underwent an MRI scan. Of these, 10 were excluded due to excessive motion artefacts, processing errors or missing sequences, 101 children (49 (49%) boys; median age, 9.5 (range: 8.9 to 12.4) years) were included in the volumetric analysis and 98 in the diffusion analysis (Supplemental Fig. 1).

Baseline characteristics of children who were and were not included in the MRI analysis were similar, except that children included in the MRI analysis were born at a higher gestation, less likely to be a twin, and less likely to have preterm as their primary risk for hypoglycaemia, reflecting the eligibility criteria for the MRI study of birth at ≥ 36 weeks (Supplemental Table 1).

In the volumetric cohort, the median gestational age at birth was 38.1 weeks (range: 36.0 to 42.7 weeks) (Table 1). A total of 20 (19%) were born preterm (< 37 weeks), 54 (54%) were born early term (37 to 38 weeks), 19 (19%) were born full-term (39 to 40 weeks), and 8 (8%) were born late-term (41 to 42 weeks). The median birth weight was 3100 (range: 1610 to 5200) g, and head circumference at birth was 34.5 (range: 29.0 to 40.0) cm. Most were singleton (94%), infants of a diabetic mother (55%), and were of European descent (60%). These characteristics were similar across the neonatal hypoglycaemia exposure groups (Supplemental Table 2).

**Table 1:** Demographic characteristics of the study cohort.

Characteristic	Children with volumetric data
N	101
Maternal characteristics	
Smoking during pregnancy^¶^	26 (27)
Alcohol consumption during pregnancy^¶^	9(9)
Education level	
Schooling incomplete	5 (6)
High school ≥ 3 years	20 (24)
Technical or trade	30 (35)
University	30 (35)
Neonatal characteristics	
Boys	49 (49)
Gestational age at birth, weeks	38.3 (1.5)
Preterm (< 37 weeks)	20 (19)
Early term (37 to 38 weeks)	54 (54)
Full-term (39 to 40 weeks)	19 (19)
Late term (41 to 42 weeks)	8 (8)
Birthweight, g	3212 (872)
Birthweight z-score	0.09 (1.80)
Head circumference at birth, cm^¶^	34.4 (2.3)
Head circumference at birth z-score^¶^	0.20 (1.64)
Twin	6 (6)
Afebrile seizures^¶^	2 (2)
Risk factors for hypoglycaemia^‡^	
Infant of diabetic mother	49 (49)
Large	12 (12)
Small	17 (17)
Preterm	17 (17)
Other	6 (5)
High deprivation^†^	12 (32)
Child characteristics	
Age at the time of MRI scan, years	9.4 (0.3)
Ethnicity	
Māori	35 (35)
Pacific	3 (3)
Asian	2 (2)
European	61 (60)

Data are presented as n (%) or mean (standard deviation) unless otherwise specified.

^¶^Missing data: smoking: 5; alcohol: 7; education level: 19; head circumference at birth: 32; head circumference at birth z-score: 32; afebrile seizures: 10.

^‡^Small: < 10th centile or < 2.5 kg; large: > 90th centile or > 4.5 kg; others: sepsis, haemolytic disease of the newborn, respiratory distress, congenital heart disease, and poor feeding.

^†^High deprivation = New Zealand deprivation index 8 to 10.

Similar characteristics were also found in the diffusion cohort.

### Brain volumes

**Figure 1:**
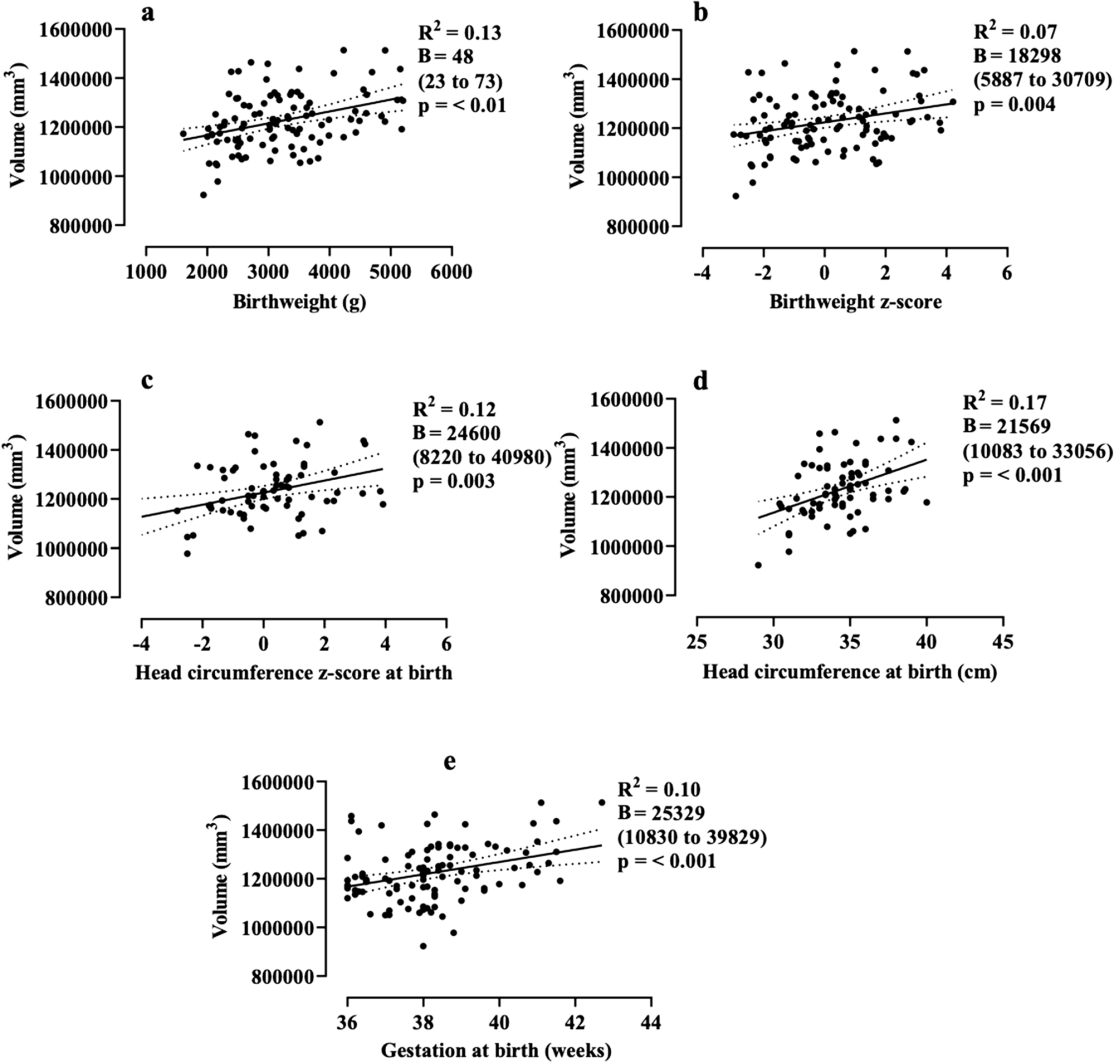
Relationship between birthweight (**a,b**), head circumference at birth (**c,d**), and gestation at birth (**e**), and total brain volume at 9 to 10 years of age. Data are linear regressions with 95% confidence intervals shown as dotted lines. B = absolute beta coefficient (95% confidence interval).

At 9 to 10 years, gestation at birth (absolute beta coefficient (B) = 25,329 mm^3^/week; 95% CI, 10,830–39,829), absolute birthweight (48mm^3^/g; 95%CI, 23–73), birthweight z-score (18,298 mm^3^; 95%CI, 5887–30,709), absolute head circumference at birth (21,569 mm^3^/cm; 95% CI, 10,083–33,056), and head circumference at birth z-score (24,600 mm^3^; 95%CI, 8220–40,980) were positively associated with total brain volume (Table 2; Fig. 1). Gestation at birth, birthweight (absolute and z-score), and head circumference at birth (absolute and z-score) were also positively associated with volumes of the total cortical grey matter, cerebral white matter, subcortical grey matter, frontal, parietal, and temporal lobes at 9 to10 years.

**Table 2:** Univariate analysis of gestation at birth, birthweight, and head circumference at birth on brain volumes at 9 to10 years of age.

Volume (mm^3^)	Gestation at birth (weeks)		Birthweight				head circumference at birth			
			Absolute (g)		z-score		Absolute (cm)		z-score	
	R^2^	B^†^ (95% CI), mm^3^/week	R^2^	B^†^ (95% CI), mm^3^/g	R^2^	β^†^ (95% CI)	R^2^	B^†^ (95% CI), mm^3^/cm	R^2^	B^†^ (95% CI)
Total brain	0.10	25,329*(10,830, 39,829)	0.13	48* (23,73)	0.07	18,298* (5887, 30,709)	0.17	21,569* (10,083, 33,056)	0.12	24,600* (8220, 40,980)
Cortical grey matter	0.09	11,020* (4280, 17,760)	0.10	19* (8,31)	0.06	7416* (1629, 13,203)	0.15	8977* (3666, 14,287)	0.10	10,374* (2857, 17,891)
Cerebral white matter	0.11	11,412* (4962, 17,861)	0.12	21* (10,32)	0.07	7926* (2385, 13,467)	0.13	8867* (3482, 14,253)	0.09	9964* (2329, 17,600)
Subcortical grey matter	0.09	1008* (400, 1616)	0.10	1* (0.7,2)	0.06	691* (170, 1213)	0.15	846* (360, 1332)	0.08	855* (157, 1553)
Frontal lobe	0.09	7141* (2474, 11,808)	0.09	12* (4,20)	0.05	4640* (634, 8646)	0.11	5349* (1656, 9042)	0.07	6084* (883, 11,285)
Parietal lobe	0.10	5954* (2548, 9359)	0.13	11* (5,17)	0.08	4368* (1457, 7279)	0.19	5346* (2658, 8033)	0.13	6179* (2340, 10,018)
Temporal lobe	0.09	3364* (1305, 5423)	0.16	7* (4,11)	0.11	3134* (1420, 4848)	0.20	3317* (1709, 4925)	0.15	4021* (1737, 6304)
Occipital lobe	< 0.01	818 (−807, 2444)	0.003	0.8 (−2,3)	< 0.01	65 (−1311, 1442)	< 0.01	270 (−1039, 1579)	< 0.01	153 (−1656, 1963)
Cerebellum	0.02	1891 (−344, 4127)	0.07	5* (1,9)	0.05	2250* (392, 4106)	0.14	2824* (1117, 4532)	0.10	3303* (892, 5714)
Brainstem	0.09	434* (169, 699)	0.05	0.54* (0.07,1)	0.01	158 (−74, 391)	0.09	291* (70, 512)	0.03	252 (−61, 567)

^†^Absolute beta coefficient (95% confidence interval).

^#^Partial coefficient (95% confidence interval).

*p ≤ 0.05.

However, the correlation coefficients for all these associations were weak (R^2^ 0.05 to 0.20).

When we investigated these relationships separately in boys and girls; similar relationships were found between gestation at birth, birthweight, and head circumference at birth, and brain volumes at 9 to 10 years, and the interaction terms were not significant.

In Model 1, both gestation at birth and birthweight-z score were independently associated with the total brain, cerebral white matter, parietal lobe, and temporal lobe volume (Table 3). Gestation at birth, but not birthweight z-score, was associated with cortical and subcortical grey matter, frontal lobe, and brainstem volume, whereas birthweight z-score, but not gestation, was associated with cerebellum volume.

**Table 3:** Multivariate analysis of gestation at birth, birthweight, and head circumference at birth on brain volumes at 9 to10 years of age.

Volume (mm^3^)	Model 1					Model 2				
	R^2^	Gestation at birth (weeks)		Birthweight z-score		R^2^	Gestation at birth (weeks)		Head circumference at birth z-score	
		B^#^ (95% CI), mm^3^/week	*p*	B^#^ (95% CI)	*p*		B^#^ (95% CI), mm^3^/week	*p*	B^#^ (95% CI)	***p***
Total brain	0.14	20,795 (5903, 35,688)	**0.006**	13,244 (697, 25,792)	**0.03**	0.13	11,923 (−7571, 31,418)	0.22	22,744 (6139, 39,348)	**0.008**
Cortical grey matter	0.12	9251 (2283, 16,219)	**0.009**	5168 (−703, 11,038)	0.08	0.11	4561 (−4416, 13,538)	0.31	9664 (2018, 17,310)	**0.01**
Cerebral white matter	0.15	9488 (2850, 16,125)	**0.005**	5620 (28, 11,212)	**0.04**	0.11	5745 (−3336, 14,825)	0.21	9070 (1335, 16,804)	**0.02**
Subcortical grey matter	0.13	841 (214, 1469)	**0.009**	487 (−42, 1016)	0.07	0.16	979 (174, 1784)	**0.01**	702 (17, 1388)	**0.04**
Frontal lobe	0.11	6056 (1215, 10,897)	**0.01**	3168 (−910, 7247)	0.12	0.08	2625 (−3600, 8851)	0.40	5675 (372, 1097)	**0.03**
Parietal lobe	0.15	4862 (1369, 8356)	**0.006**	3186 (242, 6129)	**0.03**	0.17	3853 (−668, 8375)	0.09	5579 (1727, 9430)	**0.005**
Temporal lobe	0.17	2499 (423, 4575)	**0.02**	2526 (777, 4275)	**0.005**	0.17	1560 (−1160, 4282)	0.25	3777 (1459, 6095)	**0.002**
Occipital lobe	0.01	868 (−837, 2575)	0.31	−145 (−1583, 1291)	0.84	0.01	−1003 (−3166, 1160)	0.35	310 (−153, 2152)	0.73
Cerebellum	0.06	1223 (−1078, 3524)	0.29	1952 (14, 3891)	**0.04**	0.10	740 (−2156, 3636)	0.61	3188 (721, 5654)	**0.01**
Brainstem	0.09	415 (137, 692)	**0.003**	57 (−176, 291)	0.62	0.12	460 (98, 821)	**0.01**	181 (−126, 489)	0.24

Model 1: gestation and birthweight z-score; Model 2: gestation and birth head circumference z-score.

^#^Partial coefficient (95% confidence interval).

*p ≤ 0.05.

Significant values are in bold.

In Model 2, head circumference z-score at birth, but not gestation at birth, was associated with volumes of the total brain, cortical grey matter, cerebral white matter, cerebellum, frontal, parietal, and temporal lobe, whereas gestation at birth, but not head circumference z-score at birth, was associated with brainstem volume (Table 3).

All partial coefficients for these associations were weak (R^2^ 0.09 to 0.17).

### Diffusion metrics

**Figure 2:**
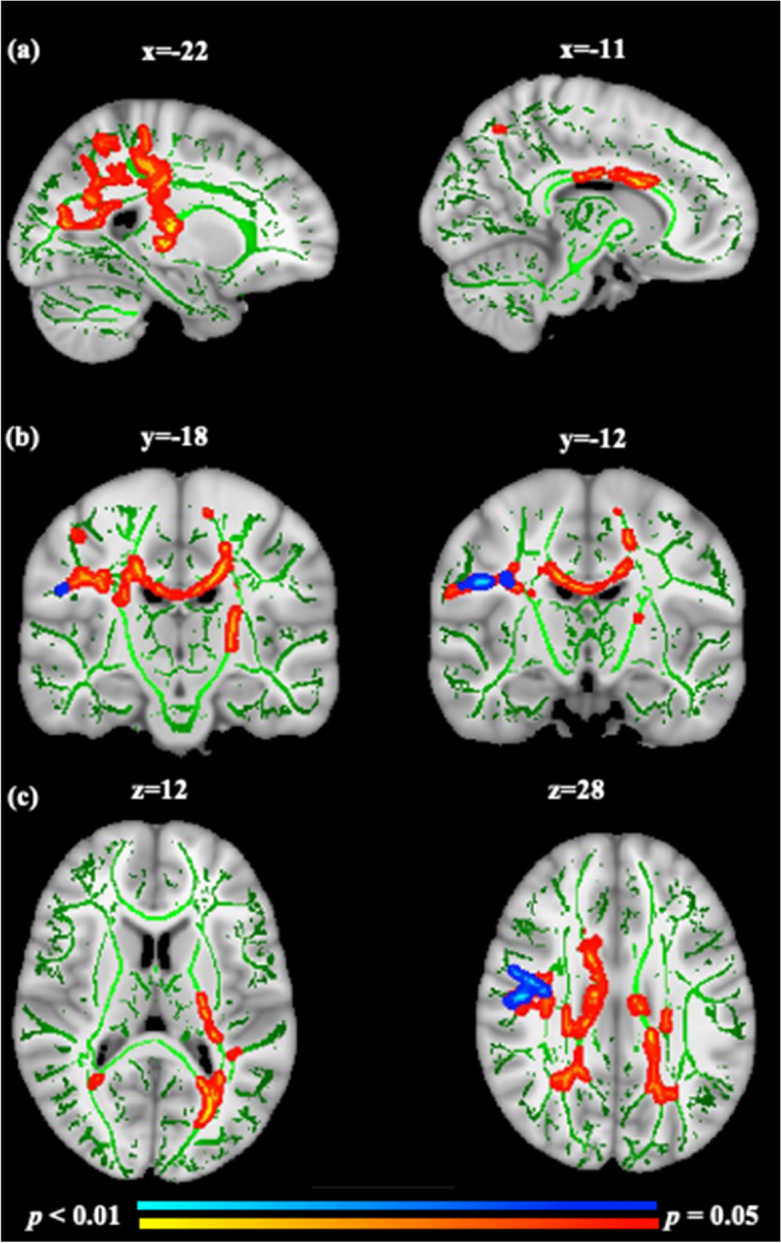
Birthweight z-scores are negatively associated with FA (red) and positively associated with RD (blue) clusters in the white matter skeleton at 9 to 10 years of age. Significant clusters are overlaid on the Montreal Neurological Institute (MNI) T1-weighted template at 1-mm thickness and on the white matter skeleton (green). Red to yellow or blue to light blue indicates the size of the *p* values after correction for multiple comparisons using Threshold-Free Cluster Enhancement (TFCE). Images are sagittal (**a**), coronal (**b**), and axial (**c**) planes and displayed in radiological convention in which the right side of the brain is left on the image. For visualisation purposes, clusters in the whole brain skeleton with significant differences were thickened using tbss_fill towards the full width of the white matter tracts.

At 9 to 10 years, gestation at birth and absolute birthweight were not associated with any diffusion metrics (FA, AD, MD, and RD) of white matter skeleton overall or in boys and girls separately, and the sex interaction terms were not significant. However, the birthweight z-score was negatively associated with FA and positively associated with RD in the right superior longitudinal fasciculus and right temporal part of the superior longitudinal fasciculus (Fig. 2). Birthweight z-score was also negatively associated with FA in the left inferior longitudinal fasciculus and left inferior fronto-occipital fasciculus.

### Post-hoc analysis

We further investigated the relationship between birthweight z-score and FA of white matter skeleton separately in children of diabetic mothers and children with other risk factors for hypoglycaemia (small or large birthweight, and preterm). We found that a lower birthweight z-score was associated with FA of white matter skeleton in the left inferior longitudinal fasciculus and inferior fronto-occipital fasciculus in children with other risk factors for hypoglycaemia but not in children of diabetic mothers, but the interaction terms were not significant (p-value for interaction = 0.61).

## Discussion

In this prospective cohort study of children born at 36 to 42 weeks’ gestation, we found that shorter gestation at birth, lower birthweight, and smaller head circumference at birth were associated with smaller brain volumes both globally and regionally at 9 to 10 years. Interestingly, both birthweight and head circumference at birth were associated with later brain volumes in these children, even after taking gestation at birth into consideration. Although gestation at birth was not associated with any of the diffusion metrics of the white matter skeleton, lower birthweight z-score was associated with higher FA, particularly in long association fibres. These associations were similar in girls and boys. Overall, these findings indicate that, even amongst those born late preterm and at term, size at birth has a persisting influence on later brain development.

We found an association between gestation at birth and brain volumes at 9 to 10 years, consistent with previous studies reporting a similar association between gestation at birth and brain volumes during mid-childhood^5,6^. Our findings support the likely benefit of longer gestation, even across the last few weeks of pregnancy, for the fetal central nervous system, where much of the maturation takes place during the third trimester, including dendritic arborization, synaptic development, axonal outgrowth, glial formation, neuronal circuitry, and connectivity formation^1^. Fetal MRI studies have also shown a positive association between gestational weeks and brain volumes over the period from 18 to 39 weeks^21^. Taken together, our findings likely reflect the fact that intrauterine influences, such as oxygenation, circulation, maternal and placental hormones, and growth factors^22^, which are important for fetal brain growth during the third trimester, are abruptly interrupted at birth, even in children born at 36 to 42 weeks’ gestation.

We also found an association between birthweight and brain volumes at 9 to 10 years, which is consistent with previous studies reporting an association between lower birthweight and smaller brain volumes during childhood and adolescence^4^. Interestingly, these relationships remained significant, although weak, even when gestation at birth was taken into consideration. Previous studies have hypothesised numerous factors, such as placental factors, maternal nutritional status, stress, ethnicity, age, body weight, parity, and environmental exposures during pregnancy^23,24^, that could be associated with poor growth of the fetus in the in-utero environment. Our findings suggest that growth before birth might have a small but persisting influence on later brain volumes at 9 to 10 years in addition to that of gestation at birth.

Head circumference at birth is considered an indicator of brain development and is widely considered an index of brain weight over the entire course of pregnancy. Our results demonstrated a positive association between head circumference at birth and brain volumes at 9 to 10 years, consistent with earlier studies showing a strong relationship between head circumference at birth and brain volumes during infancy and early childhood^7,8^. Interestingly, when we combined gestation at birth and head circumference at birth z-score in the same model, only head circumference at birth z-score remained significant and independently associated with total and most of the regional brain volumes studied at 9 to 10 years of age, suggesting that brain growth before birth, in particular, has a small but persisting influence on later brain volumes. Additionally, our findings suggest that head circumference is the strongest predictor at birth for later brain volumes in children.

We expected a positive association between the gestation at birth and birthweight and FA of white matter skeleton, as measured by diffusion imaging at 9 to 10 years. However, we found no association between gestation at birth and diffusion metrics of the white matter skeleton, consistent with an earlier study showing no association between gestational age at birth and FA of the white matter skeleton in 8-year-old children born at term^25^. Although the reason for the finding is unclear; it might reflect the postnatal acceleration of white matter after birth. Earlier studies have shown that white matter structures are immature at birth and are surrounded only by premyelin sheaths. Myelination of white matter begins from 54 postconceptional weeks onwards and increases rapidly during the first postnatal year, then continues until late adolescence^26,27^. Overall, our results suggest that white matter development that takes place postnatally may not be disrupted by being born late preterm or early term.

Contrary to our hypothesis, lower birthweight z-score was associated with higher FA and lower RD of the white matter skeleton at 9 to 10 years in long association fibres (i.e., superior longitudinal fasciculus, inferior longitudinal fasciculus, and inferior fronto-occipital fasciculus). To our knowledge, none of the earlier studies have studied this association between birthweight z-score and white matter maturation in mid-childhood in children born at ≥ 36 weeks. FA is a measure of the intravoxel directionality of white matter anisotropy, whereas RD measures diffusion perpendicular to the white matter fibres and is associated with myelin injury and/or decreased myelination^28^. In normal brain development, FA generally increases with age from childhood to adulthood in most of the white matter fibres, whereas RD generally decreases with age, because the water content in the extracellular space decreases, resulting in a decrease in RD, while the relative increase in the intracellular water content is restricted perpendicular to the myelin membranes, but not parallel to them, causing an increase in FA^29^. Previous studies in adults born small for gestation age at term, and in adults born preterm and very low birthweight, have reported lower FA in short-and long-association fibres compared to healthy controls born at term^3^. Therefore, we expected lower birthweight z-score to be associated with lower FA and higher RD of white matter skeleton. However, in our cohort, most of the large babies were born to diabetic mothers. A study in children of diabetic mothers has reported abnormal white matter microstructure at 33 and 36 weeks of gestational age, especially in the deep white matter regions, compared to children of non-diabetic mothers^30^. For this reason, we carried out a post-hoc analysis and found a negative association between birthweight and FA of white matter skeleton only in children with other risk factors but not in children of diabetic mothers. Therefore, any potential effects of maternal diabetes on offspring white matter microstructure are unlikely to explain our findings. Alternatively, given the number of analyses, our results could be due to type-1 error.

The findings of this study have some clinical significance. Firstly, longer gestation, even amongst those born at term, has potential benefits for later brain development. Secondly, this study emphasises the importance of prenatal growth for later brain development, both globally and regionally. Earlier studies have reported fetal ‘brain-sparing’ and rapid postnatal catch-up growth, particularly in head size, in children with intrauterine growth restriction^31^. Our findings suggest that this “brain sparing” effect is incomplete and that even mild degrees of intrauterine growth restriction are associated with smaller brain size to mid-childhood. Lastly, head circumference at birth is the strongest predictor of later brain volumes in children, at least to mid-childhood.

This is one of the first studies to investigate the association between specific perinatal factors and brain development and maturation at 9 to 10 years in children born late-preterm and at term. We included a large sample of children who had standardised anthropometric measurements at birth. However, this analysis has some potential shortcomings. It was an observational study on a subset of children from a large prospective cohort study, and few data were available regarding the many potential confounding prenatal and postnatal influences on brain development such as nutritional status, educational training, home environment, sleep, physical activity, etc. Although we were unable to take these factors into account in our analysis, they generally might be expected to weaken the relationships between perinatal events and later brain development. Thus our findings that these relationships are weak but still detectable at 9 to 10 years of age emphasise the importance of prenatal events for later brain development. In addition, all our included children were born at risk of neonatal hypoglycaemia and therefore, our findings cannot be generalised to other children born late preterm and at term.

Gestational age and size at birth are both associated with later brain development, even across late preterm and term gestations, suggesting that both growth before birth and duration of gestation are important for later brain development.

## Supplementary Information

Supplementary Information.

## Data Availability

All the data are available to approved researchers under the data sharing arrangements provided by the Clinical Data Research Hub, based at the Liggins Institute, University of Auckland (https://wiki.auckland.ac.nz/researchhub). Metadata, along with instructions for data access, are available at the University of Auckland’s research data repository, Figshare (https://auckland.figshare.com). Data access requests are to be submitted to Data Access Committee via researchhub@auckland.ac.nz. Deidentified published data will be shared with researchers who provide a methodologically sound proposal and have appropriate ethical and institutional approval. Researchers must sign and adhere to the Data Access Agreement that includes a commitment to using the data only for the specified proposal, to refrain from any attempt to identify individual participants, to store data securely, and to destroy or return the data after completion of the project. The Clinical Data Research Hub reserves the right to charge a fee to cover the costs of making data available if required.
