# Supplementary material for: Size at birth predicts later brain volumes
Source: Sci Rep. 2023 Aug 1;13:12446. doi: 10.1038/s41598-023-39663-9 (PMC10393952; doi:10.1038/s41598-023-39663-9)
Supplement: Supplementary file 1 — Supplementary Information. [file 41598_2023_39663_MOESM1_ESM.docx]

**
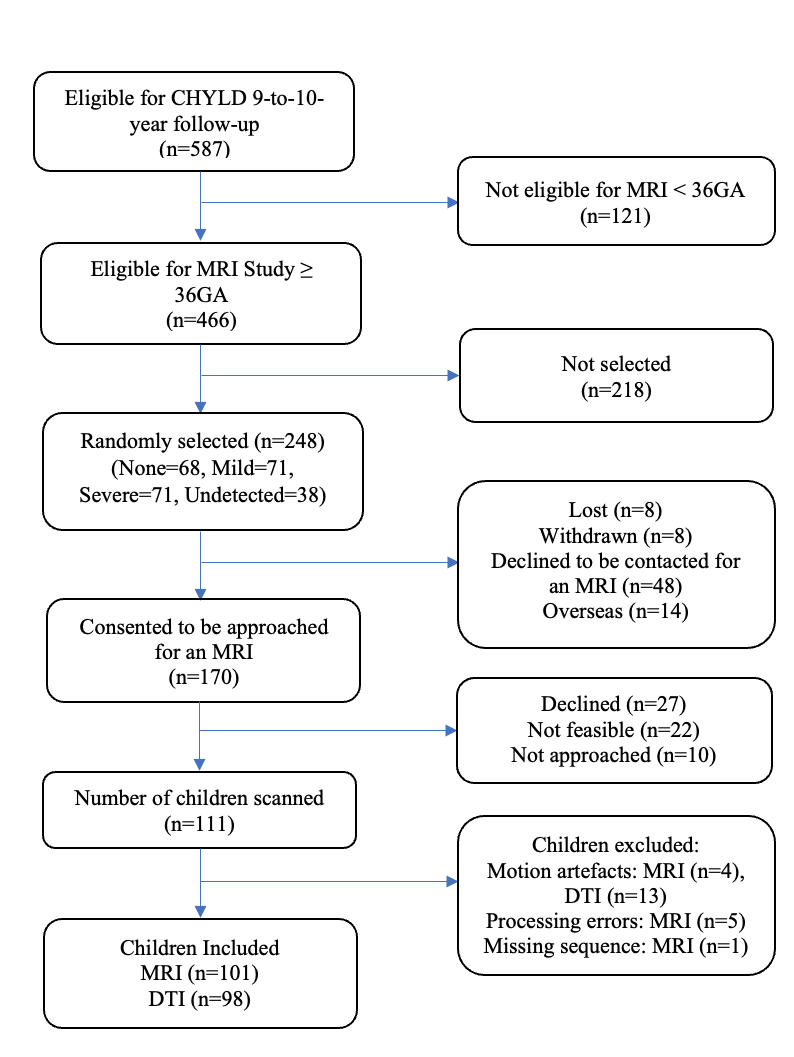
**

**Figure 1** STROBE diagram of the study population. None; no evidence of any hypoglycaemia, mild; 1 to 2 hypoglycaemic events 2.0 to 2.6mM, severe; any hypoglycaemia events <2mM or ≥ 3 hypoglycaemic events, clinically undetected hypoglycaemia; interstitial episodes only. MRI = magnetic resonance imaging, DTI = diffusion tensor imaging.

**Table 1** Characteristics of CHYLD study participants who were and were not included in the MRI analysis

| **Variable** | **All other CHYLD Mid-Childhood Outcome Study participants** | **Participants included in the MRI analysis** | ***p*** |
| --- | --- | --- | --- |
| N | 335 | 101 |  |
| Boys | 201 (53) | 49 (49) | 0.42 |
| Gestational age, weeks | 37.1 (2.2) | 38.3 (1.5) | **< 0.001** |
| Birth weight z-score | 0.08 (1.62) | 0.09 (1.80) | 0.94 |
| Birth head circumference z-score^¶^ | 0.13 (1.49) | 0.20 (1.64) | 0.76 |
| Twin | 77 (20) | 6 (6) | **< 0.001** |
| Afebrile seizures^¶^ | 11 (4) | 2 (2) | 0.55 |
| Primary risk for hypoglycaemia^‡^ |  |  | **< 0.001** |
| IDM | 126 (33) | 49 (49) |  |
| Preterm | 153 (41) | 17 (17) |  |
| Small | 54 (14) | 17 (17) |  |
| Large | 35 (9) | 12 (12) |  |
| Others | 11 (3) | 6 (5) |  |
| High deprivation^†^ | 158 (42) | 33 (33) | 0.07 |
| Age at the time of MRI (y) | 9.4 (0.4) | 9.4 (0.3) | 0.20 |
| Ethnicity |  |  | 0.45 |
| Māori | 127 (37) | 35 (35) |  |
| Pacific | 16 (3) | 3 (3) |  |
| Asian | 18 (5) | 2 (2) |  |
| European | 184 (55) | 61 (60) |  |
| Maternal education level^¶^ |  |  | 0.84 |
| Schooling incomplete | 27 (9) | 5 (6) |  |
| High school ≥3 y | 74 (23) | 20 (24) |  |
| Technical or trade | 114 (36) | 30 (35) |  |
| University | 101 (32) | 30 (35) |  |

Data are presented as n (%) or mean (standard deviation) unless otherwise specified.  ^¶^Missing data: Birth head circumference z-score: Non-participants 90, Participants 32; Afebrile seizures: Non-participants 68, Participants 13; Education level: Non-participants 63, Participants 16.

**Table 2** Baseline characteristics of participants who experienced different severities of neonatal hypoglycaemia

| **Variable** | **None** | **Mild** | **Severe** | **Undetected** | **p** |
| --- | --- | --- | --- | --- | --- |
|  |  |  |  |  |  |
| N | 26 | 24 | 30 | 21 |  |
| Boys | 13 (50) | 10 (42) | 13 (43) | 12 (57) | 0.71 |
| Gestational age, weeks | 38.4 (1.4) | 38.3 (1.6) | 38.1 (1.6) | 38.4 (1.6) | 0.89 |
| Birth weight (g) | 3230 (942) | 3393 (699) | 3135 (982) | 3097 (823) | 0.66 |
| Birth weight z-score | 0.09 (1.94) | 0.55 (1.42) | -0.03 (2.00) | -0.23 (1.74) | 0.50 |
| Birth head circumference, cm^¶^ | 34.3 (2.0) | 34.7 (1.5) | 34.4 (2.9) | 34.2 (1.9) | 0.94 |
| Birth head circumference z-score^¶^ | -0.08 (1.42) | 0.55 (1.13) | 0.31 (2.05) | -0.01 (1.60) | 0.68 |
| Twin | 1 (4) | 1 (4) | 2 (7) | 2 (10) | 0.84 |
| Afebrile seizures^¶^ | 1 (5) | 0 | 1 (5) | 0 | 0.66 |
| Primary risk for hypoglycaemia^‡^ |  |  |  |  | 0.78 |
| IDM | 12 (46) | 15 (63) | 11 (37) | 11 (52) |  |
| Preterm | 3 (12) | 4 (17) | 7 (23) | 3 (14) |  |
| Small | 6 (23) | 1 (4) | 6 (20) | 4 (19) |  |
| Large | 4 (15) | 3 (12) | 3 (10) | 2 (10) |  |
| Others | 1 (4) | 1 (4) | 3 (10) | 1 (5) |  |
| High deprivation^†^ | 9 (35) | 4 (17) | 9 (30) | 11 (52) | 0.58 |
| Age at the time of MRI (y) | 9.5 (0.6) | 9.6 (0.4) | 9.8 (0.8) | 9.6 (0.4) | 0.27 |
| Ethnicity |  |  |  |  | 0.46 |
| Māori | 7 (27) | 8 (33) | 10 (33) | 10 (48) |  |
| Pacific | 2 (8) | 0 | 0 | 1 (2) |  |
| Asian | 1 (4) | 0 | 1 (3) | 0 |  |
| European | 16 (61) | 16 (67) | 19 (64) | 10 (48) |  |
| Maternal education level^¶^ |  |  |  |  | 0.84 |
| Schooling incomplete | 1 (3.8) | 1 (4.1) | 2 (6.6) | 1 (4.7) |  |
| High school ≥3 y | 5 (19.2) | 4 (16.3) | 7 (30.0) | 4 (19.0) |  |
| Technical or trade | 7 (26.9) | 8 (33.3) | 8 (26.7) | 7 (33.3) |  |
| University | 7 (26.9) | 10 (41.7) | 8 (26.7) | 5 (23.8) |  |

Data are presented as n (%) or mean (standard deviation). IDM, Infant of diabetic mother. None, no evidence of hypoglycaemic events; Mild, 1 or 2 hypoglycaemic events between 2.0 to 2.6mM; Severe, any hypoglycaemic events < 2mM or ≥ 3 hypoglycaemic events; Undetected, interstitial episodes only. Hypoglycaemic events are defined as the sum of nonconcurrent hypoglycaemic and interstitial episodes more than 20 minutes apart. A hypoglycaemic episode is defined as at least one consecutive blood glucose concentration (BGC) < 2.6mM. Interstitial episodes are defined as at least 10 minutes of interstitial glucose concentrations < 2.6mM. ^¶^Missing data: Birth head circumference: None 5, Mild 10, Severe 5, Undetected 12; Afebrile seizures: None 4, Mild 4, Severe 2, Undetected 4; Maternal education: None 6, Mild 1, Severe 3, Undetected 4. ^‡^Small: < 10^th^ centile or < 2.5kg; Large: > 90^th^ centile or > 4.5kg; Others: sepsis, haemolytic disease of the newborn, respiratory distress, congenital heart disease, and poor feeding. ^†^High deprivation: NZDPI 8 to 10.
